# Iron Deposition Characteristics of Deep Gray Matter in Elderly Individuals in the Community Revealed by Quantitative Susceptibility Mapping and Multiple Factor Analysis

**DOI:** 10.3389/fnagi.2021.611891

**Published:** 2021-04-14

**Authors:** Jing Li, Qihao Zhang, Yena Che, Nan Zhang, Lingfei Guo

**Affiliations:** ^1^Department of Radiology, Beijing Friendship Hospital, Capital Medical University, Beijing, China; ^2^Department of Radiology, Weill Cornell Medical College, Cornell University, New York City, NY, United States; ^3^Department of Clinical Laboratory, Shandong Provincial Hospital Affiliated to Shandong First Medical University, Jinan, Shandong, China; ^4^Department of Radiology, Shandong Provincial Hospital Affiliated to Shandong First Medical University, Jinan, Shandong, China

**Keywords:** quantitative susceptibility mapping, multiple factor analysis, magnetic resonance imaging, iron deposition, deep gray matter

## Abstract

**Purpose:**

The objective of this study was to determine which factors influence brain iron concentrations in deep gray matter in elderly individuals and how these factors influence regional brain iron concentrations.

**Methods:**

A total of 105 elderly individuals were enrolled in this study. All participants underwent detailed magnetic resonance imaging (MRI) examinations from October 2018 to August 2019. Among them, 44 individuals had undergone a previous MRI examination from July 2010 to August 2011. Quantitative susceptibility mapping (QSM) was utilized as an indirect quantitative marker of brain iron, and the susceptibility values of deep gray matter structures were obtained. Univariate analysis and multiple linear regression analysis were used to investigate 11 possible determinants for cerebral iron deposition.

**Results:**

Our results showed no sex- or hemisphere-related differences in susceptibility values in any of the regions studied. Aging was significantly correlated with increased insusceptibility values in almost all analyzed brain regions (except for the thalamus) when we compared the susceptibility values at the two time points. In a cross-sectional analysis, the relationship between gray matter nucleus susceptibility values and age was conducted using Pearson’s linear regression. Aging was significantly correlated with the susceptibility values of the globus pallidus (GP), putamen (Put), and caudate nucleus (CN), with the Put having the strongest correlations. In multiple linear regression models, associations with increased susceptibility values were found in the CN, Put, red nucleus, and dentate nucleus for individuals with a history of type 2 diabetes mellitus (T2DM). However, the patients with hypertension showed significantly reduced susceptibility values in the red nucleus and dentate nucleus. Our data suggested that smokers had increased susceptibility values in the thalamus. No significant associations were found for individuals with a history of hypercholesterolemia and Apolipoprotein E4 carrier status.

**Conclusion:**

Our data revealed that aging, T2DM, and smoking could increase iron deposition in some deep gray matter structures. However, hypertension had the opposite effects in the red nuclei and dentate nuclei. Brain iron metabolism could be influenced by many factors in different modes. In future studies, we should strictly control for confounding factors.

## Introduction

Iron is the most abundant trace element in the human body. Iron in the nervous system is also involved in many fundamental biological processes, including oxygen transportation, DNA synthesis, catecholamine neurotransmitters, and myelin formation. Iron homeostasis is needed to maintain normal physiological brain function, whereas dysregulation of iron homeostasis can cause neurotoxicity through different mechanisms (Ward et al., 2014). Abnormal increases in iron content have been reported to be associated with many neural diseases, such as Parkinson’s disease, Alzheimer’s disease (AD), and Huntington’s disease (Wu et al., 2012), but restless leg syndrome is characterized by reduced iron concentrations in the substantia nigra (SN) (Rizzo and Plazzi, 2018). In Parkinson’s disease, an increase in iron concentration is noted in the SN (Faucheux et al., 2003); however, pantothenate kinase-associated neurodegeneration is characterized by an excess of iron mainly in the globus pallidus (GP), leading to a typical MRI pattern called the eye of the tiger (Kruer et al., 2011). These facts illustrate that brain iron patterns are characteristic of disease or disease stages; therefore, investigating the physiological distribution of iron in the normal brain is very important to better understand the disease-related changes that involve iron deposition.

In addition to aging, many studies have revealed that total iron concentrations increase with age in the SN, putamen (Put), GP, caudate nucleus (CN), and cortex (Xu et al., 2008; Ramos et al., 2014), but few studies have focused specifically on elderly individuals. Age-related accumulation of iron might be an important factor that contributes to neurodegenerative processes. Because many neurodegenerative disorders involve elderly people, we selected elderly individuals in the community as our target population in this study. We expect our results to present some constructive suggestions for future studies to better investigate the complex pathophysiological processes underlying neurological disorders. Previous MRI studies that focused on differences in brain iron levels between sexes reported inconsistent findings. Whereas one study reported lower iron levels in the thalamus (Thal) and red nucleus (RN) in females than in males (Gong et al., 2015), another study did not observe any difference (Xu et al., 2008). The other objective of the present study was to detect the effects of sex on regional brain iron concentrations. Many elderly people have histories of hypertension, diabetes mellitus (DM), or hypercholesterolemia. Cerebral microbleeds (CMBs), white matter hyperintensities (WMHs), and cerebral microinfarcts are also common brain MRI findings of elderly people (Valdés Hernández et al., 2016; van Veluw et al., 2017; Haller et al., 2018). Furthermore, we estimated the influence of these factors on brain iron levels in this study.

The emergence of the quantitative susceptibility mapping (QSM) technique over the past decade (Wang and Liu, 2015) has enabled accurate and reproducible *in vivo* measurements of local brain iron levels under both normal and pathological conditions (Acosta-Cabronero et al., 2016; Wang et al., 2017). Many neurodegenerative disorders with brain iron accumulation are usually associated with excess iron accumulation in deep gray matter (DGM) (Schipper, 2012), and DGM plays a crucial role in regulating movements and in various types of learning (Seger, 2008). In addition, DGM has the highest iron content (Peterson et al., 2019), and susceptibility values calculated from QSM have been reported to have a strong correlation with iron concentration in the human brain, especially in DGM (Hinoda et al., 2015). Accordingly, the objective of this study was to utilize QSM as a quantitative marker of brain iron to detect possible factors influencing the iron levels of the DGM [Thal, CN, Put, GP, SN, RN, and dentate nucleus (DN)] to accurately map iron in elderly individuals in the community, which might help us to better control confounding factors in future research.

## Materials and Methods

### Participants

The volunteers of this study were recruited from the community. The participants signed an informed consent form approved by the Institutional Review Board of Shandong Medical Imaging Research Institute Affiliated to Shandong University (ID: 2018-002). Subjects were excluded if they had a history of neurological disease or neurological ailment, including immune, metabolic, toxic, and infectious diseases or head trauma. If the subjects had other diseases, such as malignant tumors, renal insufficiency, alcoholism, or autoimmune disease, they were excluded. All MR images were evaluated by an experienced neuroradiologist for signs of space-occupying lesions and cerebrovascular diseases. Subjects with evidence of infarct (except for lacunar infarcts) or cerebral hemorrhages (except for CMBs) were excluded from further analysis.

The final sample consisted of 105 participants (57 females and 48 males) with a mean age of 65.26 ± 6.33 years (range 50–80) and a mean education level of 11.39 ± 2.57 years (range 6–16). The history of diabetes, hypertension, hypercholesterolemia, and smoking status was taken from the participants’ self-reported medical history. Individuals with a history of type 2 DM (T2DM) or hypertension were referred to as diagnosed with T2DM or hypertensive individuals who were taking hypoglycemic drugs or antihypertensive drugs when an MRI scan was conducted. In our study, 59 (56.19%) participants had hypertension, 23 (21.90%) participants had T2DM, 32 (30.48%) participants had hyperlipidemia, and 26 (24.76%) participants smoked (details are shown in Table 1).

**TABLE 1:** Description of demographic characteristics and risk factors [mean ± SD or n (%)].

Variable		Participants	Participants
		(had one MR scan)	(had two MR scans)
No. of participants		105	44
Age (years)		65.25 ± 6.33	59.19 ± 5.31
Education (years)		11.38 ± 2.57	11.91 ± 2.64
Gender	M	48 (45.71)	22 (50.00)
	F	57 (54.29)	22 (50.00)
Hypertension	No	46 (43.81)	12 (27.27)
	Yes	59 (56.19)	32 (72.73)
Diabetes	No	82 (78.10)	31 (70.45)
	Yes	23 (21.90)	13 (29.55)
Hyperlipidemia	No	73 (69.52)	27 (61.36)
	Yes	32 (30.48)	17 (38.64)
Smoking	No	79 (75.24)	34 (77.27)
	Yes	26 (24.76)	10 (22.73)
WMHs	No	31 (29.52)	28 (63.64)
	Yes	74 (70.48)	16 (36.36)
Lacunes	No	89 (84.76)	43 (97.73)
	Yes	16 (15.24)	1 (2.27)
CMBs	No	83 (79.05)	30 (68.18)
	Yes	22 (20.95)	14 (31.82)
E4 gene	No	88 (86.27)	38 (86.36)
	Yes	14 (13.73)	6 (13.64)

*Participants who had one MR examination were referred to as individuals who had an MRI brain scan from October 2018 to August 2019. Participants who had two MR examinations were referred to as individuals who had MRI scans at two time points: the first was from July 2010 to December 2011, the second was from October 2018 to August 2019, and the time interval was approximately 8 years. The Apolipoprotein E4 (APOE4) gene carrier status of 102 patients was determined by using blood samples obtained from October 2018 to August 2019. Individuals with E4 alleles were coded as Yes, and individuals without E4 alleles were coded as No. CMBs, cerebral microbleeds; WMHs, white matter hyperintensities.*

Among these 105 participants, 44 individuals had undergone another MRI scan in our hospital between July 2010 and December 2011, thereby providing us with an opportunity to study the age-related accumulation of brain iron using a longitudinal approach (details in Table 1). During the research, we fully considered the influence of gadolinium intake on the susceptibility values in the brain. We carefully reviewed the clinical and imaging data of 44 participants, and we followed up by telephone patients who underwent two MRI examinations. None of these patients had an enhanced MRI scan with gadolinium contrast agent.

### MRI Acquisition

From October 2018 to August 2019, all subjects were imaged on a MAGNETOM Skyra 3.0 T MR scanner (Siemens Healthcare, Erlangen, Germany) using a product 32-channel head coil for signal reception. The brain scanning protocol consisted of a 3D T1-weighted (T1W) magnetization-prepared rapid gradient-echo (MPRAGE) sequence for anatomic structures [repetition time (TR) = 2,300 ms, echo time (TE) = 2.3 ms, inversion time (TI) = 900 ms, flip angle = 9°, and isotropic voxel size = 1 mm^3^] and a 3D multiecho gradient-echo (ME-GRE) sequence for QSM (TR = 50 ms, first TE = 6.8 ms, TE interval = 4.1 ms, number of echoes = 10, flip angle = 15°, and voxel size = 1 mm × 1 mm × 2 mm). In addition, T2-weighted (T2W) turbo spin-echo images (TR = 3,700 ms, TE = 109 ms, TI = 900 ms, flip angle = 15°, and slice thickness = 5 mm), T2W fluid-attenuated inversion recovery (FLAIR) images (TR = 8,000 ms, TE = 81 ms, TI = 2,370 ms, flip angle = 15°, and slice thickness = 5 mm), diffusion-weighted images (DWIs) (TR = 3,700 ms, TE = 65 ms, flip angle = 18°, and slice thickness = 5 mm), and susceptibility-weighted images (SWIs) (TR = 27 ms, TE = 20 ms, flip angle = 15°, and slice thickness = 5 mm) were acquired to detect brain abnormalities.

From July 2010 to December 2011, 44 participants were evaluated on a Signa 3.0T MRI scanner (Signa, HDx, General Electric Healthcare, Milwaukee, WI, United States) with an eight-channel array coil. The scanning sequences included conventional MRI sequences (T2W imaging, T2-FLAIR, and DWI), 3D T1W imaging for anatomic structure, and enhanced 3D multiecho GE T2^∗^-weighted angiography (ESWAN) sequences for QSM. 3D T1W images were acquired using a T1W volumetric fast spoiled gradient recalled-echo (FSPGR) sequence with the following parameters: TR = 7.3 ms, TE = 2.7 ms, TI = 850 ms, flip angle = 13°, and isotropic voxel size = 1 mm^3^; ESWAN sequences were acquired using a 3D-enhanced T2^∗^-weighted contrast flow-compensated (i.e., the gradient moment was null in all three orthogonal directions) ME-GRE (12 different TEs) sequence with the following parameters: TR = 51.6 ms, first TE = 4.4 ms, TE = 4.4–38.2 ms, TE interval = 4.8 ms, number of echoes = 8, flip angle = 20°, and voxel size = 1 mm × 1 mm × 2 mm.

### Quantitative Susceptibility Mapping Preprocessing and Quantitative Analysis

**FIGURE 1:**
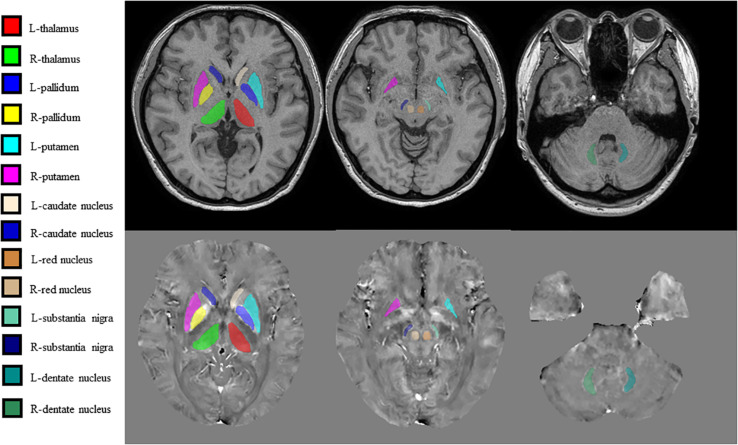
Regions of interest (ROIs) were traced directly on the quantitative susceptibility mapping (QSM) image and T1-weighted (T1W) images to remove veins with high positive susceptibility values on QSM.

Brain QSM maps were computed from ME-GRE complex image data using morphology-enabled dipole inversion with an automatic uniform cerebrospinal fluid (CSF) zero reference algorithm (MEDI + 0) (Liu et al., 2018). Briefly, a non-linear fitting of the multiecho data was performed to estimate the total field, followed by spatial field unwrapping and background field removal using the projection onto dipole fields (PDF) algorithm to compute the local field (Liu et al., 2011), which was then inverted to obtain the final susceptibility map. Structural priors (edges) within the lateral ventricles derived from the magnitude image and with a regularization term enforcing a uniform susceptibility distribution of the CSF were used in the numerical inversion to improve QSM quality and to provide CSF as an automatic susceptibility reference. The CSF mask was determined by thresholding the R2^∗^ map computed from the ME-GRE magnitude data and imposing voxel connectivity (Liu et al., 2018). The conventional images (T1W, T2W, and FLAIR) were processed with an automated FMRIB Software Library (FSL) pipeline, which consisted of brain data extraction using the Brain Extraction Tool (BET) algorithm (Smith, 2002), bias field correction using FMRIB’s Automated Segmentation Tool (FAST) (Zhang et al., 2001), and linear coregistration to the ME-GRE magnitude image (which is in the same space as QSM) using FMRIB’s Linear Registration Tool (FLIRT) with six degrees of freedom (Jenkinson et al., 2002). For the region of interest (ROI) analysis, FMRIB’s Integrated Registration Segmentation Tool (FIRST) (Patenaude et al., 2011) was used to segment selected subcortical GM structures (Thal, CN, Put, GP, RN, SN, and DN) on T1W images, and the resulting segmentation masks were linearly coregistered to QSM. These masks were then visually inspected and manually edited by an experienced neuroradiologist if necessary (e.g., to remove veins or CMBs with high positive susceptibility values) on QSM images using ITK-SNAP v3.8 software^1^. The mean susceptibility value within each ROI was recorded (Figure 1).

### Conventional MRI Assessment

According to the Standards for Reporting Vascular Changes on Neuroimaging (STRIVE) criteria (Wardlaw et al., 2013a), WMHs were signal abnormalities of variable size, defined as white matter hyperintense lesions on FLAIR images. Lacunes were round or ovoid, subcortical small lesions (3–15 mm in diameter) that were hypointense on T1W images and hyperintense on T2W images and had a perilesional halo on FLAIR images. CMBs were defined as small signal voids (≤10 mm in diameter) with associated blooming on T2^∗^-weighted images. The severity of CMBs, lacunes, and WMHs was assessed using a simple scoring method (Amin Al Olama et al., 2020). In a binary fashion (i.e., presence or absence), individuals with lacunar infarcts, WMHs, or CMBs were coded as 1; and individuals without these MRI findings were coded as 0.

### APOE Genotyping

Apolipoprotein E (APOE) genotyping was conducted using standard real-time fluorescence polymerase chain reaction (PCR) methods (Calero et al., 2009). APOE4 carrier status was determined: individuals with E4 alleles were coded as 1, and individuals without E4 alleles were coded as 0.

### Statistical Analysis

Statistical analysis was performed using Statistical Package for the Social Sciences software (Version 21.0 for Windows; SPSS, Chicago, IL, United States). The measurement data are presented as the mean ± standard deviation. The count data were represented in the form of n (%). The effect of different hemispheric locations was compared using a paired *t*-test. To test the independent association between variables and susceptibility values in DGM, we performed multiple regression analyses. Considering that our sample size is not large enough, we made variable selection before doing the multiple linear regression analysis. First, we performed a univariate analysis using an independent sample *t*-test and Pearson correlation analysis. Only variables for which a univariate association with susceptibility values at a *p*-value < 0.1 was seen were entered into the multiple regression models as independent variables (Pirpamer et al., 2016). Second, for every ROI, the multiple linear relationships between the independent parameters and the susceptibility values were estimated with a stepwise regression method, and the best multiple linear regression models were selected.

After univariate analysis, for the Thal, variables of smoking status, history of T2DM and hypertension, APOE carrier status, and whether having CMBs in the brain were introduced to the following multiple linear regression analysis. For the Put, variables of aging, history of T2DM, and whether having CMBs in the brain were introduced to the following multivariate analysis. For the CN, the variables are aging and history of T2DM; for the RN, the variables are aging, history of T2DM and hypertension, whether having lacunes in the brain; for the SN, the variables are aging and history of hypertension; for the DN, the variables are aging, gender, history of T2DM, and hypertension.

Among these 105 participants, 44 patients received two MRI examinations at two different time points. Hence, the age-related accumulation of brain iron could be analyzed longitudinally with paired *t*-tests. Because multiple hypotheses were tested, we used the Bonferroni method for correction to avoid type I errors.

## Results

For susceptibility values, no significant effect of hemispheric location in the seven ROIs was found. Therefore, in the following analysis, we used the mean values of the left and right hemispheres (Table 2).

**TABLE 2:** Comparison of the susceptibility values of the gray matter nuclei between the left and right hemispheres (mean ± SD).

	Mean value	Left	Right	*t*	*p*
Thalamus	0.04 ± 14.37	0.40 ± 14.53	0.33 ± 15.96	0.724	0.470
Globus pallidus	185.82 ± 47.47	184.08 ± 48.25	187.57 ± 49.78	–1.461	0.147
Putamen	98.79 ± 31.49	97.45 ± 33.09	100.12 ± 33.05	–1.358	0.178
Caudate nucleus	83.24 ± 22.94	83.89 ± 23.78	82.59 ± 24.13	0.967	0.336
Red nucleus	149.58 ± 42.62	148.19 ± 44.53	150.98 ± 44.36	–1.13	0.261
Substan tia nigra	158.96 ± 39.90	159.13 ± 43.10	158.78 ± 40.06	0.152	0.879
Dentate nucleus	111.16 ± 40.86	111.88 ± 42.38	110.42 ± 41.12	0.871	0.386

**FIGURE 2:**
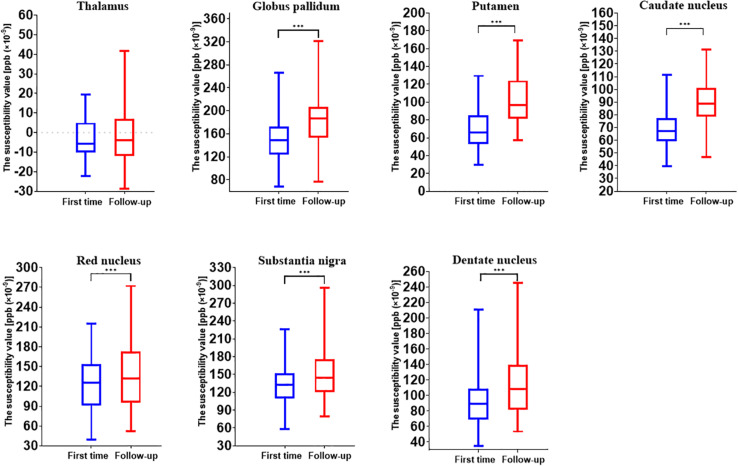
Comparison results of susceptibility values of gray matter nuclei between two time points (first time, July 2010 to December 2011; follow-up, October 2018 to August 2019). ***p < 0.001.

The longitudinal analysis of 44 individuals who had two MRI scans showed the age-related accumulation of brain iron in the GP (*t* = 10.763, *p* < 0.001), Put (*t* = 20.657, *p* < 0.001), CN (*t* = 10.308, *p* < 0.001), RN (*t* = 3.497, *p* = 0.001), SN (*t* = 4.976, *p* < 0.001), and DN (*t* = 8.576, *p* < 0.001) but not in the Thal (*t* = 0.659, *p* = 0.513) (Figure 2).

**FIGURE 3:**
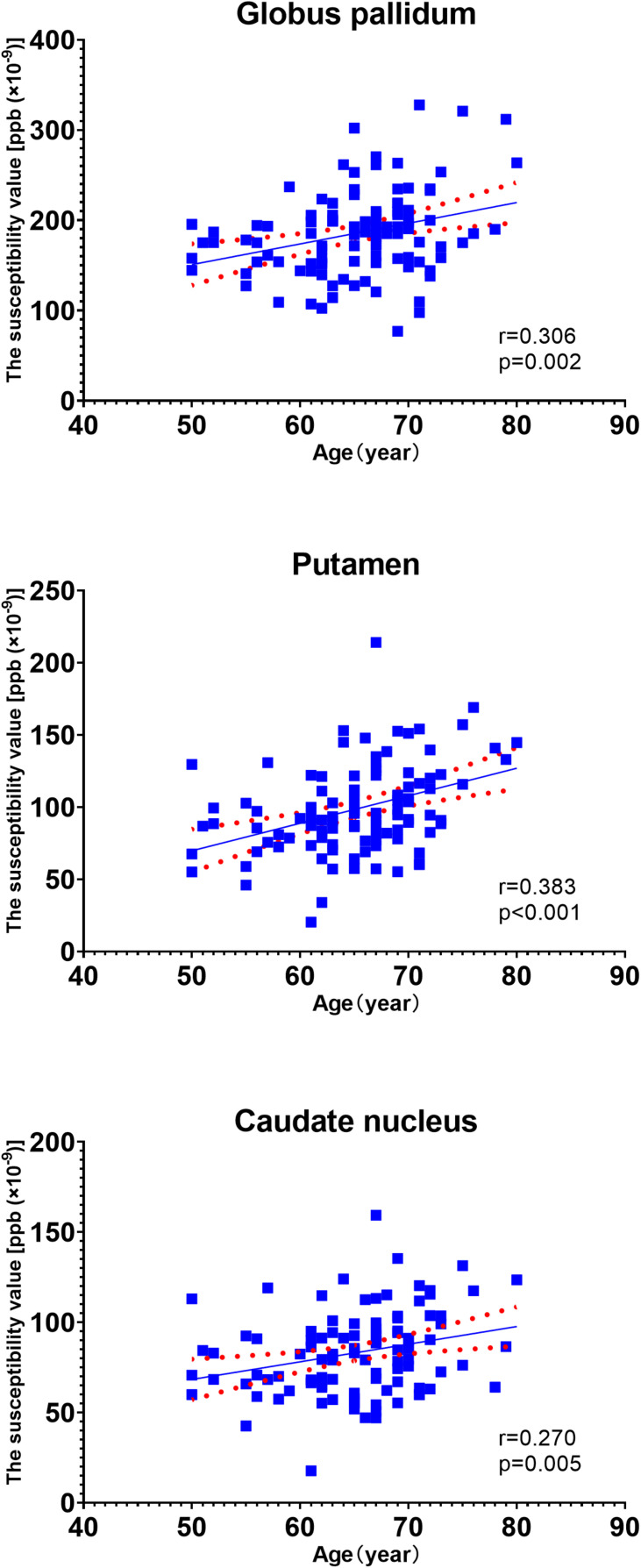
Scatter plots illustrating a linear age dependency of iron concentration as measured by mean quantitative susceptibility mapping (QSM) susceptibility values. The putamen exhibited the highest rate of increase in susceptibility values with aging.

In the univariate analysis for each region, the factors with p values less than 0.1 are highlighted in italics (Table 3). Pearson’s correlation analysis showed increased rates of susceptibility values with aging varying among these regions. Put exhibited the highest rate of increase in susceptibility with aging (Table 4). Scatter plots illustrated a linear age dependency of iron concentration as measured by mean QSM susceptibility values (Figure 3).

**TABLE 3:** Determinants of susceptibility values in gray matter structures: results of univariate analysis.

		Thalamus			Globus pallidus			Putamen			Caudate nucleus			Red nucleus			Substantia nigra			Dentate nucleus		
		Susceptibility	Statistical		Susceptibility	Statistical		Susceptibility	Statistical		Susceptibility	Statistical		Susceptibility	Statistical		Susceptibility	Statistical		Susceptibility	Statistical	
		value	value		value	value		value	value		value	value		value	value		value	value		value	value	
			*t*	*p*		*t*	*p*		*t*	*p*		*t*	*p*		*t*	*p*		*t*	*p*		*t*	*p*
CMBs	No (83)	-1.52 ± 13.60	-2.190	*0.031*	186.96 ± 48.35	0.476	0.635	95.53 ± 29.41	-2.094	*0.039*	82.33 ± 22.03	-0.786	0.434	148.94 ± 40.14	-0.301	0.794	159.58 ± 37.54	0.309	0.758	111.47 ± 42.60	0.154	0.878
	Yes (22)	5.89 ± 15.97			181.52 ± 44.77			111.09 ± 36.54			86.67 ± 26.37			152.03 ± 51.92			156.61 ± 48.73			109.95 ± 34.34		
WMH	0 (31)	0.20 ± 15.27	0.076	0.940	181.69 ± 43.50	-0.575	0.566	93.74 ± 30.00	-1.064	0.290	83.47 ± 25.15	0.068	0.946	152.68 ± 40.24	0.480	0.632	162.26 ± 38.45	0.548	0.585	109.13 ± 44.13	-0.327	0.744
	1 (74)	-0.03 ± 14.08			187.55 ± 49.21			100.90 ± 32.06			83.14 ± 22.12			148.29 ± 43.78			157.57 ± 40.66			112.00 ± 39.70		
Lacunes	0 (89)	-0.49 ± 14.84	-0.893	0.374	187.52 ± 48.55	0.865	0.389	97.49 ± 31.83	-1.000	0.320	83.25 ± 23.08	0.010	0.992	153.17 ± 41.23	2.065	*0.041*	160.87 ± 38.29	1.163	0.248	112.91 ± 42.36	1.042	0.300
	1 (16)	2.99 ± 11.36			176.36 ± 41.02			106.04 ± 29.43			83.18 ± 22.88			129.64 ± 46.03			148.29 ± 47.88			101.36 ± 30.44		
Hypertension	No (46)	2.13 ± 15.85	1.325	0.188	190.74 ± 46.77	0.936	0.351	97.61 ± 30.60	-0.338	0.736	83.78 ± 24.38	0.213	0.831	161.25 ± 36.39	2.541	*0.013*	165.95 ± 38.43	1.599	0.113	118.90 ± 44.04	1.732	*0.086*
	Yes (59)	-1.60 ± 13.00			182.00 ± 48.05			99.71 ± 32.41			82.81 ± 21.95			140.49 ± 45.14			153.50 ± 40.49			105.11 ± 37.47		
Diabetes	No (82)	-1.33 ± 14.26	-1.858	*0.066*	186.54 ± 48.46	0.292	0.711	95.54 ± 33.36	-2.024	*0.046*	80.21 ± 23.18	-2.623	*0.010*	145.53 ± 43.31	-1.860	*0.066*	156.87 ± 38.16	-1.014	0.313	106.12 ± 41.18	-2.440	*0.016*
	Yes (23)	4.90 ± 14.00			183.20 ± 44.64			110.36 ± 23.92			94.02 ± 18.77			164.02 ± 37.45			166.41 ± 45.93			129.10 ± 34.91		
Hyperlipidemia	No (73)	-0.15 ± 15.29	-0.199	0.842	190.50 ± 51.98	1.534	0.128	97.64 ± 33.19	-0.565	0.574	82.44 ± 23.92	-0.533	0.595	152.52 ± 42.57	1.029	0.306	161.42 ± 37.99	0.956	0.341	112.35 ± 44.07	0.451	0.653
	Yes (32)	0.46 ± 12.23			175.16 ± 33.38			101.42 ± 27.55			85.04 ± 20.77			143.12 ± 42.69			153.33 ± 44.07			108.42 ± 32.85		
E4 gene	No (88)	-0.94 ± 14.05	-1.792	*0.076*	185.64 ± 48.92	-0.229	0.819	99.30 ± 29.95	-0.274	0.785	83.53 ± 21.37	-0.859	0.392	150.21 ± 41.33	0.208	0.835	159.67 ± 40.66	0.466	0.642	115.12 ± 41.81	1.619	0.109
	Yes (14)	6.48 ± 16.46			188.82 ± 35.75			101.75 ± 37.98			89.00 ± 26.77			147.62 ± 53.22			154.23 ± 40.31			96.36 ± 27.89		
Smoke	No (79)	-1.55 ± 12.81	-2.004	*0.048*	184.30 ± 43.97	-0.572	0.568	95.98 ± 28.33	-1.606	0.111	82.50 ± 20.95	-0.571	0.569	148.68 ± 41.95	-0.379	0.706	157.27 ± 36.68	-0.753	0.453	112.81 ± 43.35	0.725	0.470
	Yes (26)	4.86 ± 17.75			190.46 ± 57.54			107.33 ± 39.00			85.47 ± 28.50			152.34 ± 45.34			164.08 ± 48.88			106.10 ± 32.35		
Gender	M (48)	1.49 ± 16.18	0.949	0.345	182.50 ± 52.27	-0.657	0.513	97.19 ± 35.03	-0.476	0.635	81.67 ± 24.39	-0.641	0.523	146.74 ± 40.93	-0.626	0.533	161.60 ± 44.49	0.622	0.533	103.47 ± 41.56	-1.786	*0.077*
	F (57)	-1.19 ± 12.66			188.62 ± 43.29			100.14 ± 28.43			84.56 ± 21.77			151.98 ± 44.21			156.73 ± 35.84			117.62 ± 39.47		

*Significant *p*-values < 0.10 are highlighted in italics. CMBs, cerebral microbleeds; WMH, white matter hyperintensity.*

**TABLE 4:** The Pearson linear relationship between susceptibility values of DGM and age.

	Thalamus	Globus pallidus	Putamen	Caudate nucleus	Red nucleus	Substantia nigra	Dentate nucleus
*r*	–0.060	0.306	0.383	0.270	0.112	0.113	0.221
*p*	0.570	0.002	0.000	0.005	0.257	0.252	0.024

*The Bonferroni method was used for multiple hypothesis correction to avoid type I error, and the new *p*-value level was adjusted to 0.007. DGM, deep gray matter.*

Because the longitudinal analysis showed that age played a key role in susceptibility values in the DGM (except for the Thal), age was introduced into the subsequent multiple linear regression analysis. After the multiple linear regression analysis, we found that the influencing factor for Thal was smoking status (*t* = 2.166, *p* = 0.033). Regarding Put, CN, RN, and DN, the influencing factors included a history of T2DM (*t* = -3.094, *p* = 0.003; *t* = 2.682, *p* = 0.009; *t* = 2.069, *p* = 0.041; and *t* = 2.682, *p* = 0.009, respectively) and age (*t* = 4.257, *p* = 0.000; *t* = 2.897, *p* = 0.005; *t* = 3.164, *p* = 0.001; and *t* = 3.126, *p* = 0.002, respectively). In these regions, the results showed that T2DM patients had increased iron deposition. Age-related increases in susceptibility values could also be detected, and the rates of increase with aging varied among these regions. For RN and DN, a history of hypertension also influenced the susceptibility values (*t* = −3.236, *p* = 0.002; *t* = −2.818, *p* = 0.006, respectively), and patients with hypertension showed significantly lower iron content in these regions. Details of the multiple linear regression models are listed in Table 5.

**TABLE 5:** Determinants of susceptibility values in gray matter structures: results of multiple linear stepwise regression analysis.

	Factors	*r*	Adjusted *r*	*T*	*p*	*R* of model	*F* of model	*P* of model
Thalamus	Smoking	7.120	0.212	2.166	0.033	0.212	4.692	0.033
Putamen	Age	1.895	0.381	4.257	0.000	0.428	11.447	0.000
	Diabetes	14.494	0.191	2.137	0.035			
Caudate nucleus	Age	0.967	0.267	2.897	0.005	0.366	7.882	0.001
	Diabetes	13.643	0.247	2.682	0.009			
Red nucleus	Age	1.533	0.310	3.164	0.001	0.392	5.170	0.000
	Diabetes	19.459	0.185	2.069	0.041			
	Hypertension	–27.122	–0.317	–3.236	0.002			
Dentate nucleus	Age	1.917	0.297	3.126	0.002	0.409	6.657	0.000
	Diabetes	23.972	0.244	2.682	0.009			
	Hypertension	–21.968	–0.268	–2.818	0.006			

We analyzed interaction effects by introducing three new factors: age × history of hypertension, age × history of DM, and history of hypertension × history of DM. In the Put, CN, and RN, no interaction effects were found. However, an interaction effect was found in the DN. Aging was associated with susceptibility values only in the participants who had no hypertension or DM (data not shown).

## Discussion

**FIGURE 4:**
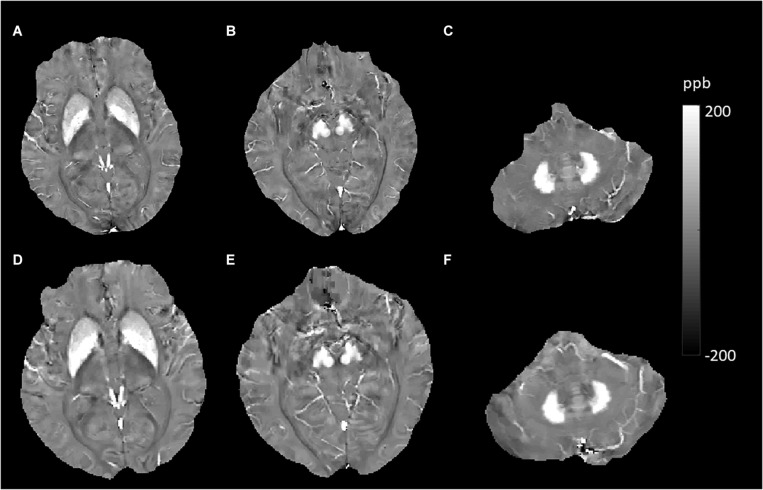
Evolution of susceptibility values with age in the caudate nucleus, putamen, globus pallidus, red nucleus, substantia nigra, and dentate nucleus. These images were from an 80-year-old male non-smoker without hypertension and type 2 diabetes mellitus (T2DM). The upper row **(A–C)** shows quantitative susceptibility mapping (QSM) images acquired in 2010, and the bottom row **(D–F)** shows QSM images acquired in 2019. Susceptibility values of the gray matter nuclei increased significantly over time.

**FIGURE 5:**
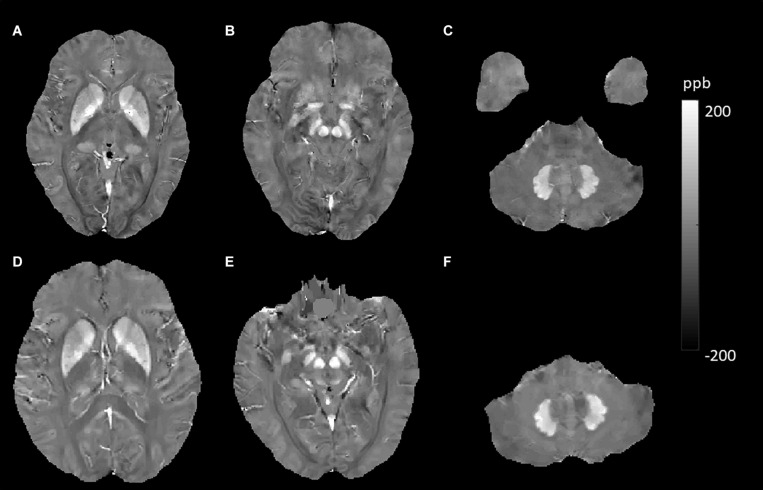
Evolution of susceptibility values with age in the caudate nucleus, putamen, globus pallidus, red nucleus, substantia nigra, and dentate nucleus. These images were from a 64-year-old female non-smoker without hypertension and type 2 diabetes mellitus (T2DM). The upper row **(A–C)** shows quantitative susceptibility mapping (QSM) images acquired in 2011, and the bottom row **(D–F)** shows QSM images acquired in 2019. Susceptibility values of the gray matter nuclei increased significantly over time.

In this study, using the QSM method, we investigated some factors and their effects on brain iron content. These factors included hemispheric location, sex, aging, smoking status, APOE carrier status, history of T2DM, hypertension, hyperlipidemia, and some important MRI features (CMBs, WMHs, and cerebral microinfarcts) in elderly people in the community. We expect that this knowledge will help us to gain more insight into the potential abnormalities of magnetic susceptibility that arise from various neurological diseases and neural degeneration. Similar to many other studies, our data indicated significant effects of age on susceptibility values in the DGM. In the cross-sectional analysis, age-related iron deposition was found in the GN, Put, CN, and DN (Xu et al., 2008; Li et al., 2014; Gong et al., 2015). Based on the knowledge that QSM is reproducible across scanner makers, models, field strengths, and sites and could be used in clinical investigations both longitudinally and across centers (Hinoda et al., 2015; Wang et al., 2017; Wicaksono et al., 2020), the MR measures of QSM were accurate and consistent across repeated measurements and between platforms (Hobson et al., 2020). We performed a longitudinal analysis of age-related iron deposition despite different scanners in the two examinations. The results showed significant age-related increases in GN, Put, CN, RN, SN, and DN but not in Thal (Figures 4, 5). Notably, the rate of increase varies among these regions, reflecting a heterogeneous accumulation of iron in different brain tissues. The highest rate of increase was found in the Put. Our results showed no support for a linear age-related change in iron content in the Thal, consistent with some previous studies (Bilgic et al., 2012; Gong et al., 2015; Persson et al., 2015). These findings illustrated that when we intend to study the iron deposition of neurological diseases involving the DGM, especially the Put; age, as a confounding factor, should be controlled more strictly.

**FIGURE 6:**
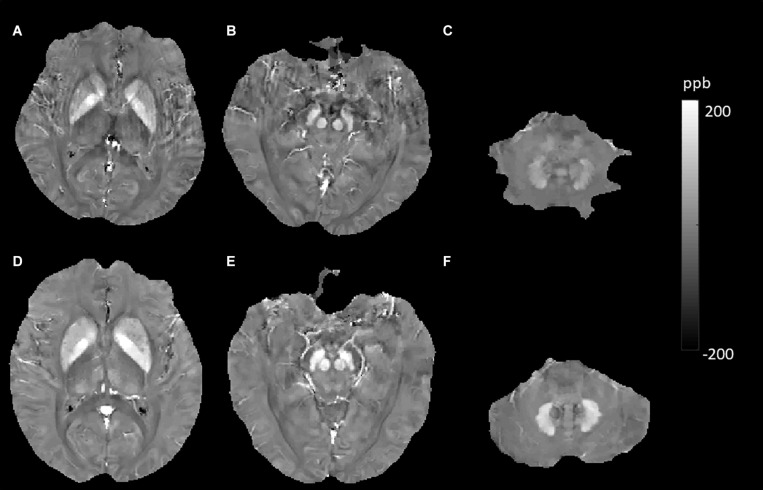
Evolution of susceptibility values with type 2 diabetes mellitus (T2DM) effect in the putamen, caudate nucleus, red nucleus, and dentate nucleus. The upper row **(A–C)** shows susceptibility images of a 55-year-old male non-smoker without hypertension and T2DM (first). The bottom row **(D–F)** shows quantitative susceptibility mapping (QSM) images of a 52-year-old male non-smoker with T2DM but without hypertension (second). Susceptibility values of the putamen, caudate nucleus, red nucleus, and dentate nucleus were higher in the second participant than in the first participant.

In this study, we found that brain iron was abnormally deposited in patients with T2DM in several DGM, including the Put, CN, RN, and DN (Figure 6). A previous study investigated the deposition of iron in the brain in T2DM patients with mild cognitive impairment using QSM and showed similar results. They found that susceptibility values in the right CN and SN and the left Put increased and that the susceptibility of these regions was closely correlated with cognitive impairment, suggesting that iron deposition may play an important role in the process of T2DM cognitive impairment (Yang et al., 2018). The precise mechanisms underlying the higher iron concentration in T2DM are not understood, but hyperglycemia could lead to neuronal damage. Neurons have a continuous high glucose demand, and unlike muscle cells, they cannot accommodate episodic glucose uptake under the influence of insulin. Neuronal glucose uptake depends on the extracellular concentration of glucose, and cellular damage can ensue after persistent episodes of hyperglycemia (Tomlinson and Gardiner, 2008). Iron accumulation could occur as an epiphenomenon of demyelination, axonal damage, and/or neurodegeneration (Raz et al., 2015). Insulin resistance leads to high permeability of the blood–brain barrier (BBB) and triggers cognitive decline in a diabetic insulin resistance mouse model and in an AD model (Takechi et al., 2017). Increased permeability with leakage of material into the vessel wall and perivascular tissue could cause inflammation (Wardlaw et al., 2013b), and the inflammatory status of the brain could influence brain iron metabolism and lead to iron deposition (McCarthy et al., 2018). Therefore, we speculate that neuronal damage caused by hyperglycemia and permeability of the BBB caused by insulin resistance may be the reasons for iron accumulation in T2DM, but this inference needs to be substantiated by more studies.

In addition to brain iron dysmetabolism in patients with hypertension, we initially speculated that the brain iron content would be increased in hypertensive patients. Conversely, we observed decreased susceptibility in the RN and DN. Many studies have suggested that increased systolic blood pressure (SBP) is associated with injury to the white matter microstructure and gray matter atrophy (Maillard et al., 2012; Fennema-Notestine et al., 2016; Carnevale et al., 2018). Few studies have examined the effect of hypertension on regional brain iron deposition. A study reported that hypertensive participants had significantly greater iron content in the hippocampus, CN, Put, entorhinal cortex, superior frontal gyrus, and primary visual cortex (Rodrigue et al., 2011). One study of cognitively impaired patients showed that DM-positive patients had lower susceptibility values, indicative of lower brain iron content, than DM-negative patients in the hippocampus and pulvinar of the Thal (Park et al., 2018); however, in that study, hypertension was more frequently found in DM-positive patients (69.6% vs. 39.1%). It was unclear whether hypertension might be the cause of the lower brain iron content that caused the mixed effects of hypertension and DM to lead to the different results. These heterogeneous results suggested that hypertension or other neurological diseases may cause complicated iron redistribution, and the white matter, cortex, and DGM may show different or completely opposite changes. This hypothesis has been verified by a study that found decreased iron levels in the temporal cortex in postmortem human brains with Parkinson’s disease (Yu et al., 2013).

**FIGURE 7:**
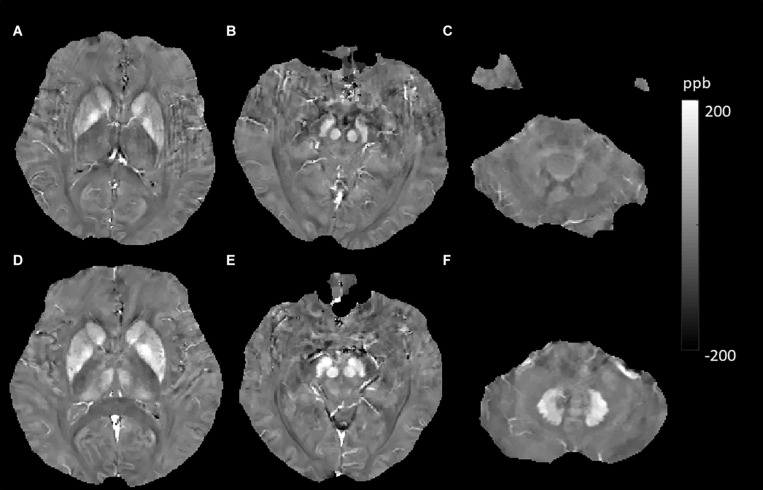
Evolution of susceptibility values with smoking effects in the thalamus. The upper row **(A–C)** shows susceptibility images of a 56-year-old male non-smoker without hypertension and type 2 diabetes mellitus (T2DM) (first). The bottom row **(D–F)** shows quantitative susceptibility mapping (QSM) images of a 55-year-old male smoker without hypertension and T2DM (second). The susceptibility value of the thalamus was higher in the second participant than in the first participant.

Our data represented that smokers had increased brain iron levels in the Thal (Figure 7). Active and passive tobacco smoking (TS) has been associated with vascular endothelial dysfunction in a causative and dose-dependent manner primarily related to the release of reactive oxygen species (ROS), and ROS can also induce cerebral inflammation (Kaisar et al., 2018). Brain inflammation and BBB impairment could cause iron accumulation, which might lead to extraordinarily increased susceptibility values in Thal smokers. A study reporting brain iron accumulation in unexplained fetal and infant death victims with mothers who were smokers also supported our results (Lavezzi et al., 2011). In the Thal, we found that individuals who carried APOE4 had higher susceptibility values (6.48 vs. -0.94). This suggested that the APOE4 gene might aggravate brain iron deposits. Previous studies suggest that APOE4 is a major genetic risk factor for AD and poor neurological outcomes after traumatic brain injury and hemorrhage (Bell et al., 2012). APOE4 could lead to accelerated BBB breakdown and thereby cause neuronal and synaptic dysfunction (Montagne et al., 2020). These findings indicate that brain iron overload in APOE4 carriers comes from destroyed cerebrovascular integrity. However, the *p*-value was 0.076 (close to 0.05) in our study. This might be because the sample of APOE4 carriers was not large enough, as only 14 subjects carried the APOE4 gene in our participants. This inference requires a larger sample size to be confirmed.

We found a slightly lower susceptibility value of the Thal than other studies, and some individuals even had negative values. We think that it may be different from the research population and related to the different ROI tracking methods. Low susceptibility in DGM was also reported in two previous QSM analyses (Kim et al., 2017; Uchida et al., 2019). These results may suffer from systematic errors in QSM reconstruction or white noise. Furthermore, all QSM images were generated using the MEDI + 0 method, which uses the value in CSF as a zero reference; this could partly eliminate the effect of systematic error and background white noise.

Previous MRI studies that focused on differences in brain iron levels between sexes and hemispheric locations reported different results (Xu et al., 2008; Gong et al., 2015; Persson et al., 2015; Chai et al., 2019; Peterson et al., 2019). However, our data showed no sex- or hemisphere-related differences in iron levels in any of the regions studied. These heterogeneous results might be caused by the differences in the characteristics of the populations studied. With more data or by using a meta-analysis (reasonably merging data from different studies), we might elucidate the sex- and hemisphere-related effects more powerful and effectively. We did not find that CMBs, WMHs, or cerebral microinfarcts influenced the brain iron levels in the studied regions; we speculated that these common brain MR findings in elderly people were caused by hypertension, T2DM, aging, or other factors related to common vascular risk factors; thus, they did not directly influence brain iron levels.

Our present study has several limitations. First, this is mainly cross-sectional research, and the sample size of the longitudinal study was relatively small. In the future, we will follow up with more individuals and provide more powerful evidence demonstrating which factors influence iron accumulation. Second, we measured iron content in brain regions that have both calcification and iron accumulation (such as the GP), which may have affected the accuracy of the iron content measurement. Third, we did not investigate white matter. Because the brain injury caused by hypertension was closely related to the white matter microstructure, iron metabolism of white matter should be evaluated in more detail in the future. Fourth, other influencing factors, such as body mass index (BMI), serum iron concentrations, and the usage of hypoglycemic drugs and antihypertensive drugs, may also induce changes in regional susceptibility values and should be investigated in the future.

## Conclusion

Our data confirmed the significant effects of age on susceptibility values in DGM. The highest rate of iron deposition with aging was observed in the Put. T2DM and hypertension had the opposite effects on iron metabolism in the DGM. Our data confirmed that smokers had increased brain iron levels in the Thal. These results showed that iron metabolism is complicated and that different diseases have different patterns. These results also indicate that in future studies, we should not only pay attention to conventional factors, such as age and sex, but also consider other confounding factors, including disease history and smoking status, to better elucidate the underlying mechanisms of iron-related neurodegenerative diseases.

## Data Availability Statement

The raw data supporting the conclusions of this article will be made available by the authors, without undue reservation.

## Ethics Statement

The studies involving human participants were reviewed and approved by the Institutional Review Board of Shandong Medical Imaging Research Institute Affiliated to Shandong University. The patients/participants provided their written informed consent to participate in this study.

## Author Contributions

JL and LG wrote the main manuscript text. QZ and YC prepared the imaging data, Figures 1–7, and the tables. NZ prepared the clinical data. LG revised the main manuscript text. All the authors reviewed the manuscript.

## Conflict of Interest

The authors declare that the research was conducted in the absence of any commercial or financial relationships that could be construed as a potential conflict of interest.

## Footnotes

1 www.itksnap.org
